# Mechanical and Durability Evaluation of Metakaolin as Cement Replacement Material in Concrete

**DOI:** 10.3390/ma15227868

**Published:** 2022-11-08

**Authors:** Mohammed Najeeb Al-Hashem, Muhammad Nasir Amin, Ali Ajwad, Muhammad Afzal, Kaffayatullah Khan, Muhammad Iftikhar Faraz, Muhammad Ghulam Qadir, Hayat Khan

**Affiliations:** 1 Department of Civil and Environmental Engineering, College of Engineering, King Faisal University, Al-Ahsa 31982, Saudi Arabia; 2 Civil Engineering Department, University of Management and Technology, Lahore 54770, Pakistan; 3 Civil Engineering Department, University of Engineering and Technology, Taxila 47050, Pakistan; 4 Department of Mechanical Engineering, College of Engineering, King Faisal University, Al-Ahsa 31982, Saudi Arabia; 5 Department of Environmental Sciences, Abbottabad Campus, COMSATS University Islamabad, Abbottabad 22060, Pakistan; 6 Department of Chemical Engineering, University of Engineering and Technology, Peshawar 25120, Pakistan

**Keywords:** pozzolans, kaolin clay, metakaolin, workability, compressive strength, water permeability, sorptivity, water absorption, acid attack

## Abstract

Due to the increasing prices of cement and its harmful effect on the environment, the use of cement has become highly unsustainable in concrete. There is a considerable need for promoting the use of cement replacement materials. This study investigates the effect of variable percentages of metakaolin (MK) on the mechanical and durability performance of concrete. Kaolin clay (KC) was used in the current research to prepare the MK by the calcination process; it was ground in a ball mill to its maximum achievable fineness value of 2550 m^2^/Kg. Four replacement levels of MK, i.e., 5%, 10%, 15%, and 20% by weight of cement, in addition to control samples, at a constant water-to-cement (w/c) ratio of 0.55 were used. For evaluating the mechanical and durability performance, 27 cubes (6 in. × 6 in. × 6 in.) and 6 cylinders (3.875 in. diameter, 2 in. height) were cast for each mix. These samples were tested for compressive strength under standard conditions and in an acidic environment, in addition to being subjected to water permeability, sorptivity, and water absorption tests. Chemical analysis revealed that MK could be used as pozzolana as per the American Society for Testing and Materials (ASTM C 618:2003). The results demonstrated an increased compressive strength of concrete owing to an increased percentage of MK in the mix with aging. In particular, the concrete having 20% MK after curing under standard conditions exhibited 33.43% higher compressive strength at 90 days as compared to similarly aged control concrete. However, with increasing MK, the workability of concrete decreased drastically. After being subjected to an acid attack (immersing concrete cubes in 2% sulfuric acid solution), the samples exhibited a significant decrease in compressive strength at 90 days in comparison to those without acid attack at the same age. The density of acid attack increased with increasing MK with a maximum corresponding to 5% MK concrete. The current findings suggest that the local MK has the potential to produce good-quality concrete in a normal environment.

## 1. Introduction

In recent years, many engineers from all over the world have devoted themselves to research activities aiming to produce economy in the cement and construction industry. In this context, they emphasized the use of cement replacement materials in construction projects. There is a substantial need to encourage the use of cementation ingredients more economical than ordinary Portland cement (OPC) bearing in mind the expensive costs of Portland cement. The usage of additional cementitious materials is the first phase in developing low-cost construction constituents to be used in emerging states. Concrete is the building material usually made use of to counter forces in compression. By adding pozzolanic materials, one can improve concrete workability, strength, durability, and resistance to permeability and cracks [1]. Rojas showed that numerous current cement blends are altered with the inclusion of admixtures, which enhances their microstructure and diminishes the concentration of calcium hydroxide by consumption with the help of a pozzolanic response. The resulting adjustment of the microstructure of bond composites enhances the mechanical properties and the useful life properties overall [2]. Antonovich et al. found that with the dissemination of fine pozzolan particles in the concrete paste, countless nucleation localities can be created for the precipitation of hydration items which would make the paste more consistent [3]. Srivastava expressed that the physical impact of the fine grains permits thick pressing inside the concrete and decreases the divider impacts on the movement zone between the aggregates and paste. The mentioned zone can be considered reinforced because of higher bond improvement between the two stages, enhancing the solid microstructure and properties. By and large, the pozzolanic impact depends on the pozzolanic response, as well as upon the filler or physical impact of the smaller particles in the mix. Consequently, pozzolan inclusion into a standard Portland bond enhances its mechanical durability in addition to strength when contrasted, in light of the interface reinforcement, with the referral paste. The physical activity of the pozzolans gives homogeneous and denser paste [4].

Sharif et al. expressed that pozzolans are utilized as a part of a solid constituent with an ordinary bond as substitution materials. Initially, the term pozzolan was related to volcanic cinders and calcined earth which would ordinarily respond, within the sight of water, with lime at encompassing temperatures. These days, this term covers all aluminous/siliceous materials which are in fine powder frames and respond with calcium hydroxide within the sight of water to shape mixes that have cementitious properties [5]. Bentz found that pozzolanic responses alter concrete’s microstructure by consumption of the discharged calcium hydroxide (CH) and generation of extra calcium silicate hydrates (C–S–H) bringing about enhanced solidness. It has been found that cement replacement materials such as fly ash, GGBS, and silica fume can replace Portland cement to some extent [6].

For Pakistan, the development of cement replacement materials is of great importance, as the country has started large irrigation projects, highway networks, and building programs as well as plans for their expansion in the near future. Most of the cement replacement materials mentioned earlier are not locally available. Thus, due to expenditure on their import and cost increases, these ultimately become costlier than cement. However, it has been seen that metakaolin (MK) conforming to ASTM C618:2003 is found locally at negligible cost. Sabir et al. explained that MK (Al_2_Si_2_O_7_) has been used as a pozzolan in high-performance concrete applications and has been commercially available since the mid-1990s. Poon et al. found that it typically contains 40–45% Al_2_O_3_ and 50–55% SiO_2_ and is considered a natural pozzolan with high reactivity [7]. Neville et al. stated that pozzolana is one of the common materials classified as cementitious, which can be natural or artificial material containing reactive silica [8]. David et al. stated that kaolin or China clay is commercial clay that is principally composed of the hydrated alumino silicate clay mineral kaolinite [9].

Siddique et al. [10] hypothesized that MK would help improve the initial mechanical properties and long-term quality properties of composite mortar/paste/concrete. Ding et al. studied and analyzed the effects of MK or SF on concrete strength, workability, resistance to chloride penetration, and shrinkage. At the kneading grades listed, MK offers better workability than SF [11]. It was concluded that the compressive strength of concrete is related to both the MK-to-binder ratio and the water-to-binder ratio. The maximum strength is obtained at a 15% replacement level for all water–binder ratios [12]. Nalawade et al. [13] stated that as the percentage of MK increases, the strength increases. The FM3525 and FM3020 combination gave better results for permeability and drying shrinkage and gave high strength. It was recommended for seashore structures and underground structures where proper pumping and adequate curing are not possible.

The previous research also concluded that optimal performance was achieved by replacing 7% to 15% of the cement with MK. Compressive strength values for concrete containing MK after 28 days can be 20% higher. A dose of 15% MK caused a decrease in workability. Increasing the proportion of MK in the concrete mix required increasing the superplasticizer dosage to ensure longer workability [14]. Justice et al. [15] concluded that with regard to workability and setting time, MK required more superplasticizer and shortened setting time of pastes as compared to control mixtures and companion silica fume mixtures. Dhinakaran et al. (2012) found that the strength increase in MK concrete is effective only at the early age of concrete and is only marginal in the long term. The increase in compressive strength for MK concrete was greater at higher w/c ratios (i.e., 0.4 and 0.5), and hence higher w/c ratios were more suitable. From the studies, an optimum percentage of MK was found to be 10% for all w/c ratios except for 0.32 and for 0.32, for which it was 15% [16]. Dong et al. [17] investigated the mechanical properties and durability of MK-incorporated mortar using different curing methods and found that the compressive strength of the Portland cement containing 10% MK after 1 day of curing is 3.18 times that of pristine plain cement mortar, and the compressive strength is comparable if Portland cement is cured for three days under the same curing conditions. Prakash et al. [18] studied the mechanical properties of concrete in which natural coarse aggregates were replaced with recycled coarse aggregate and MK and concluded that the 40% substitution of natural coarse aggregate with recycled concrete aggregates carried good results with 10% MK.

In this study, special attention was paid to studying the effects of the partial replacement of cement with MK so that we may use it efficiently and obtain the maximum benefit from it according to the local environment. A typical concrete mix ratio, 1:2:4, one part cement, two parts fine aggregate, and four parts of coarse aggregate, was used with MK for different cement substitution levels. Investigations regarding the properties of the ready-mixed and hardened concrete cast from pure (control without MK) and blended cements using MK were performed at 5%, 10%, 15%, and 20% substitutions. The results obtained give a clear picture regarding the effects of partial replacement of cement by locally available MK on the fresh and hardened properties of concrete.

## 2. Materials and Methods

### 2.1. Materials

#### 2.1.1. Cement

OPC manufactured by one of the leading brands in Pakistan “FECTO cement” was used. The physical properties of the cement used are given in Table 1, while the chemical composition of the cement is shown in Table 2.

#### 2.1.2. Fine Aggregates

Lawrencepur sand was used as fine aggregate for the experimentation. As per ASTM C136-06, sieve analysis of the fine aggregate was carried out, and the results are summarized in Table 3. The fineness modulus (FM) of sand was calculated as 2.62. As per ASTM C128-79, the specific gravity of sand was calculated to be 2.71. Water absorption of fine aggregates was determined by adopting the standard procedure prescribed in BS 812-2: 1995. The water absorption of sand was found to be 1.2%.

#### 2.1.3. Coarse Aggregate

Coarse aggregate that was used for experimentation was best-quality Margalla crush. As per ASTM C136-06 guidelines, sieve analysis of coarse aggregate was performed to confirm its standard grading (Table 4). Using ASTM C127-81, a specific gravity test was also carried out, and the specific gravity of coarse aggregate was found to be 2.68. Water absorption of the coarse aggregate was determined as 0.8% for a 24 h period.

#### 2.1.4. Water

Regular tap water from the concrete laboratory was used for research purposes. Table 5 shows the properties of the water used, which were measured using standard test methods for pH, water hardness, conductivity, and sulfate contents.

#### 2.1.5. Metakaolin

**Figure 1:**
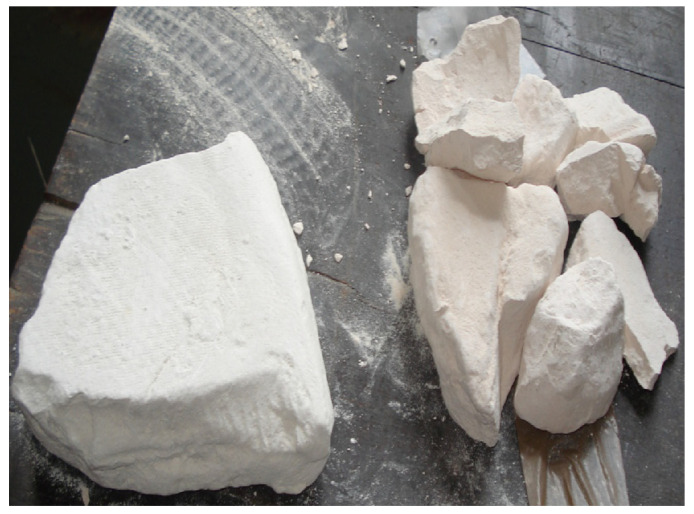
Kaolin clay (**Left**) and metakaolin (**Right**).

Best-quality kaolin clay (KC) collected from Nagar Parkar, situated in the province Sindh of Pakistan, was purchased from the local pottery/ceramics industry of Gujrat, Punjab. It was in the form of large lumps having off-white color as shown in Figure 1.

KC was transported to Glass & Ceramics Research Centre (GCRC) in the PCSIR Laboratories Complex Lahore, Pakistan, for its calcinations to obtain MK. KC was placed in the kiln for 1 h at a temperature of 800 °C. At this temperature duration, most of the absorbed water of KC was removed by the breaking of the water bond structure of KC. This disorder in the structure of KC is called dehydroxilization. This process of thermal activation of clay is termed calcination. The material obtained after heating of KC in the kiln was in the form of small lumps compared to its larger size before calcinations.

**Figure 2:**
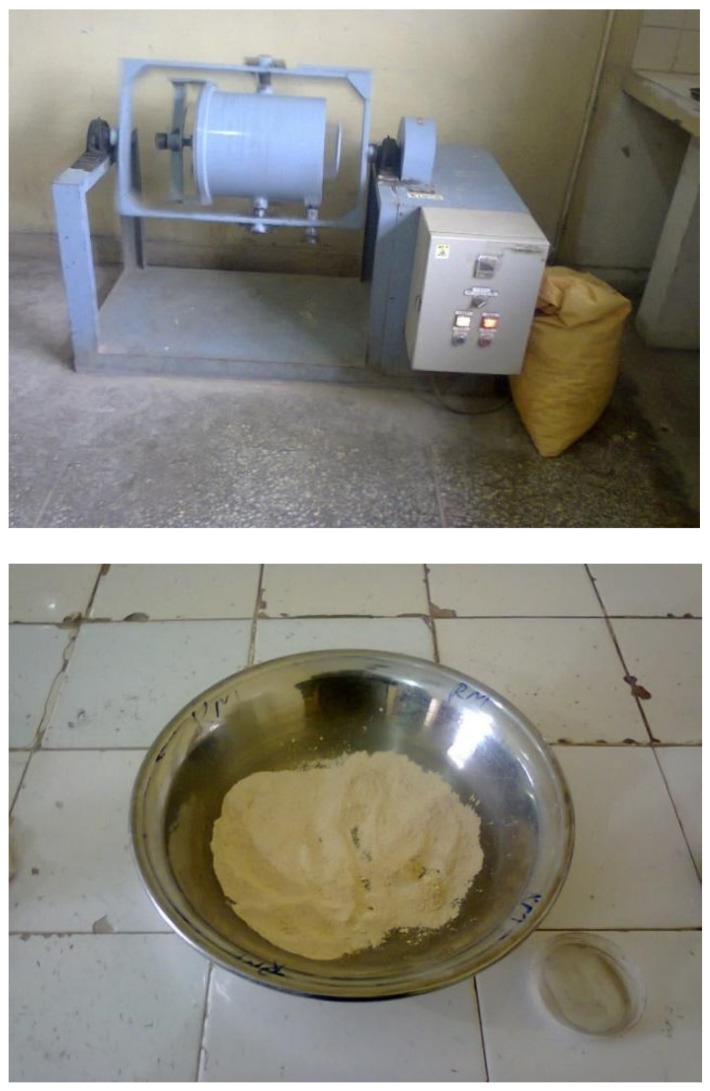
MK in ground form.

Then MK, thus produced, was transported to the physical laboratory for grinding in the ball mill to the maximum fineness, and the final ground MK produced is shown in Figure 2.

**Figure 3:**
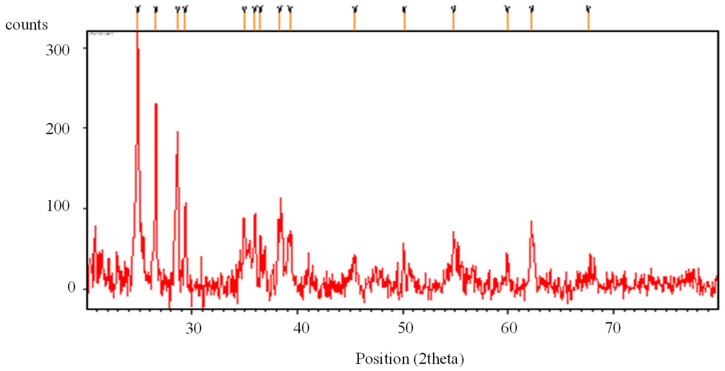
XRD pattern of MK sample.

**Figure 4:**
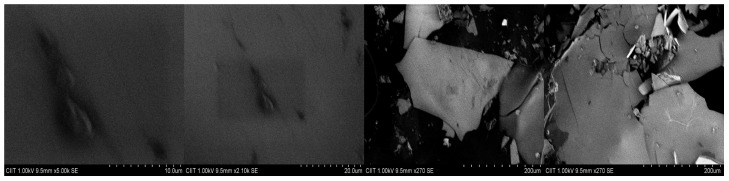
SEM images of MK sample.

XRF was used to conduct the chemical analysis of MK in its final form. Chemical analysis results presented in Table 6 demonstrate that MK has a high level of silica; i.e., the sample contains more than 70% SiO_2_, Al_2_O_3_, and Fe_2_O_3_ in the total content and thus fulfills the requirement as per ASTM C 618:2003 to be used as pozzolana. Ground MK’s XRD graph is shown in Figure 3, and the SEM images are shown in Figure 4. The XRD graph of ground MK confirms the presence of quartz, mainly in the crystalline form, whereas SEM images show that MK has a multilayered, angular, and microporous surface, which demonstrates its high specific surface area, thus showing high pozzolanic reactivity.

### 2.2. Mixing Schedule

For determining several properties of concrete, the mix ratio selected was one of the most used mix ratios for normal strength concrete, i.e., 1:2:4. A total of four replacement levels of MK by weight of cement, i.e., 5%, 10%, 15%, and 20%, having a constant w/c ratio of 0.55 were used. The motor-operated drum mixer was used to obtain a uniform mixing of concrete. The maximum capacity of the mixer was 2.5 ft^3^ per batch. All five concrete mixes without and with MK as cement replacement material are detailed in Table 7. The mix design is shown in Table 8. 

### 2.3. Casting of Concrete Specimens

Under normal curing, a total of fifteen identical cubes (6 in. × 6 in. × 6 in.) were cast for each mix to investigate the compressive strength of three identical specimens at each testing age (3, 7, 28, 56, and 90 days). In addition to normal curing, an additional six cube specimens were cast to investigate the effect of acid attack on compressive strength at the ages of 56 and 90 days. For each mix, sorptivity was investigated at 90 days of curing for three identical cylindrical samples (size 3.875 in. diameter, 2 in. height). Three identical specimens were tested to obtain average sorptivity results. Similarly, the water permeability of each mix was examined by averaging the results of three identical cube specimens (5.875 in. × 5.875 in. × 6 in.) at the ages of 28 and 56 days.

## 3. Results and Discussion

A detailed discussion of the test results related to workability, compressive strength without and with acid attack, water permeability, sorptivity, water absorption, resistance to acid attack, and density of acid-attacked concrete is given in this section.

### 3.1. Workability (Slump Test)

**Figure 5:**
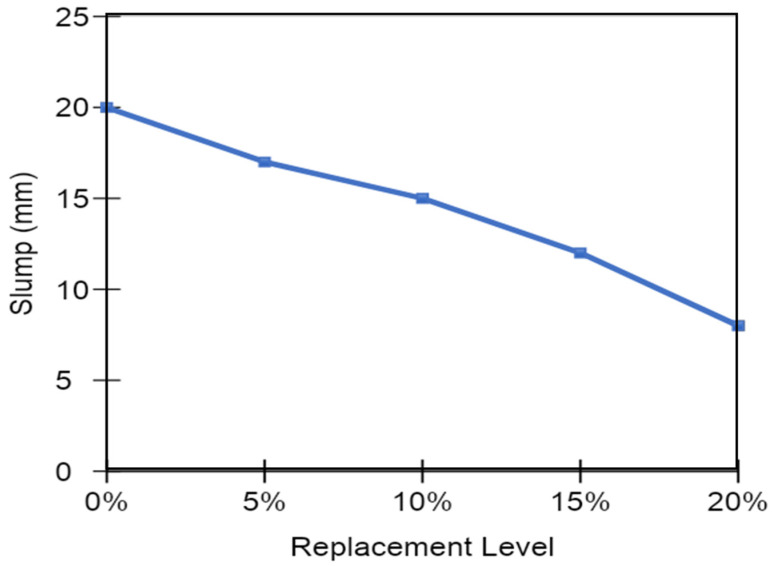
Replacement level versus slump of MK blended mixes.

The workability of various alternative grades of concrete incorporating different percentages of MK (5, 10, 15, and 20%) was recorded at constant w/c ratio and weights of cement in the mix. Figure 5 shows the comparison of slump test results between different MK concrete mixes and their comparison to the control mix. It can be seen that the workability of concrete decreased remarkably with increasing MK content in the mix. The control mix without MK showed the highest workability. Concrete having 20% MK showed a maximum loss of workability of almost 60% compared to that of the control mix. Keeping in view the decreased workability of MK mixes, the intended workability can be achieved by adding superplasticizers/water reducers or superabsorbent polymers in the concrete.

### 3.2. Compressive Strength

**Figure 6:**
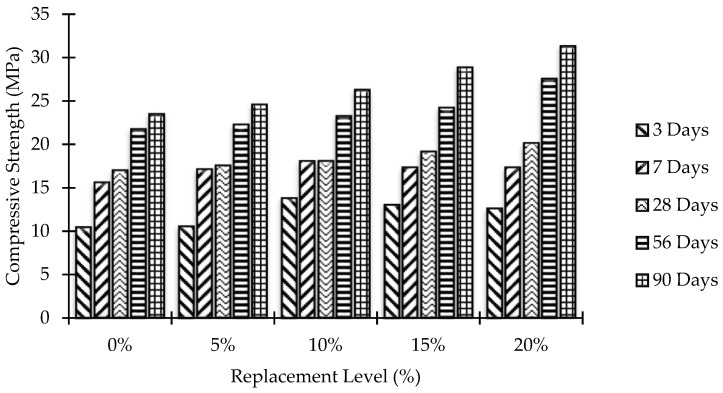
Compressive strength of MK blended mixes with aging.

For each concrete mixture, 15 out of 21 concrete cubes (6 in. × 6 in. × 6 in.) were used for normal compressive strength. After curing under standard conditions, three identical cubes were tested for each testing age, i.e., 3, 7, 28, 56, and 90 days. Thus, in total, 75 cubes were tested for normal compressive strength for all replacement levels of MK. Testing was conducted in accordance with BS 1881:1970. Figure 6 shows the results of compressive strength tests performed on samples with a typical mix ratio of 1:2:4 and a w/c ratio of 0.55 with different percentages of MK as a partial substitute for cement. The results demonstrated an increase in the compressive strength of concrete with the increasing amount of MK in the mix. At early ages (3 and 7 days), the effect of increasing MK on strength gain was insignificant. However, a remarkable increase in strength due to increasing MK can be observed at later ages (28, 56, and 90 days). The above findings suggest an increased strength due to both the increasing amount of MK and aging. Therefore, the maximum compressive strength was achieved at the highest substitution level of MK, which is 20%, at the age of 90 days. As compared to previous research [19], the replacement level for maximum compressive strength is slightly higher. This is because of the higher fineness of MK, which highly reacted with Ca(OH)_2_ and formed more calcium silicate hydrate (C–S–H), consequently resulting in higher compressive strength. In addition, the miniaturization of MK particles also contributes to increased strength. The optimal alternative MK content was found to be 20% by weight of cement.

**Figure 7:**
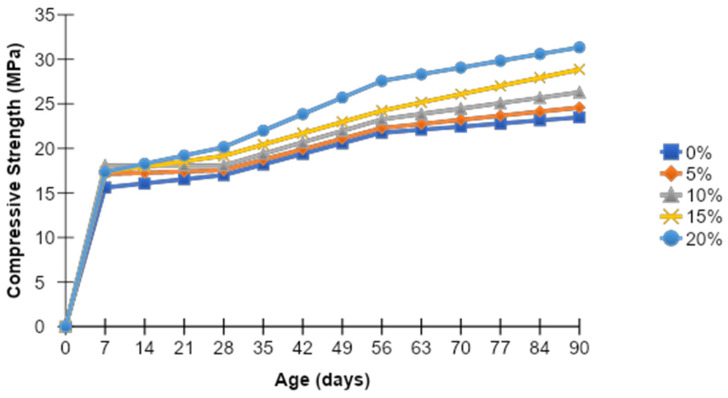
Comparison of rate of gain of compressive strength (combined) for all mixes.

Figure 7 shows a comparison of the rate of change in the compressive strength of concrete over time. It can be seen that the strength gained corresponding to 20% MK is dominant from the beginning as compared to that of the control mix. Although the trend of strength gain for 20% MK replacement with aging remained nearly constant for up to 28 days, it was still higher than the control mix. Afterward, it shows an upward trend in strength increase as compared to other replacement levels among MK mixes. Compared to the compressive strength of the control mix, the compressive strength of concrete at 20% MK substitution level increases by up to 33.43%.

### 3.3. Strength Activity Index

ASTM C 311-07 supports the estimation of the strength activity index (SAI) to assess the pozzolanic behavior of concrete. This is done by replacing predetermined bonds with pozzolans to ensure mortar quality. Test results are affected by the conditions of the concrete used, especially its fineness and soluble base cement. There is also a lime-based pozzolanic activity index that determines the total pozzolanic activity. In the present case, the SAI for MK blended concrete mixes has been calculated by the following equation:

SAI = A/B × 100(1)

where:

A = Normal compressive strength of MK blended concrete;

B = Normal compressive strength of OPC concrete (control concrete).

**Figure 8:**
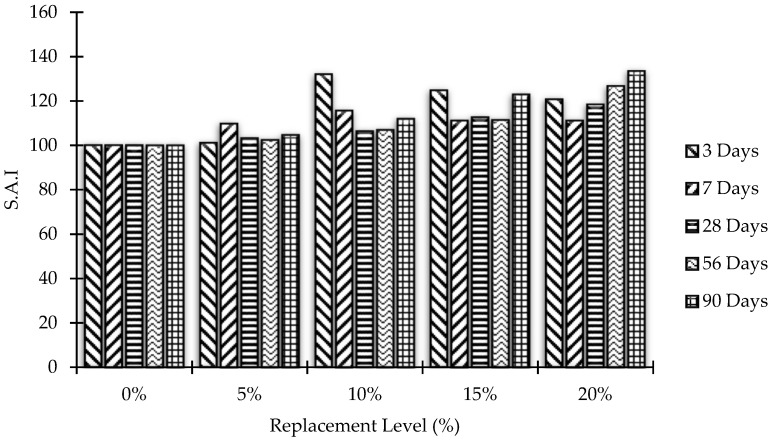
Compressive strength activity index (SAI) versus MK content at all testing ages.

**Figure 9:**
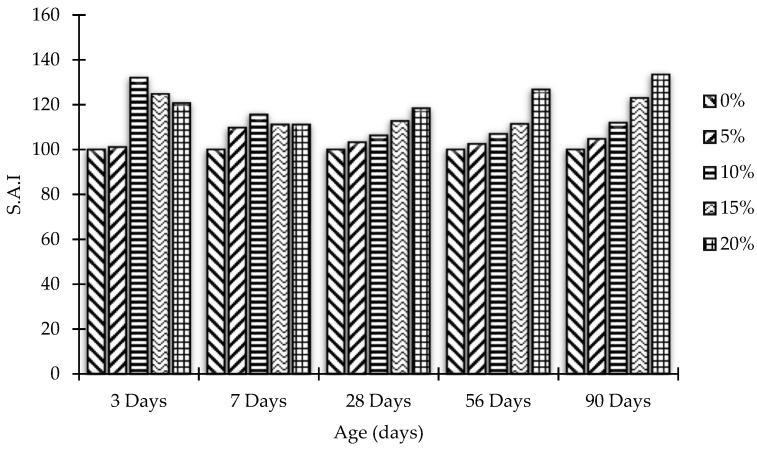
Effects of the curing age on compressive strength activity index (SAI) of all mixes.

**Figure 10:**
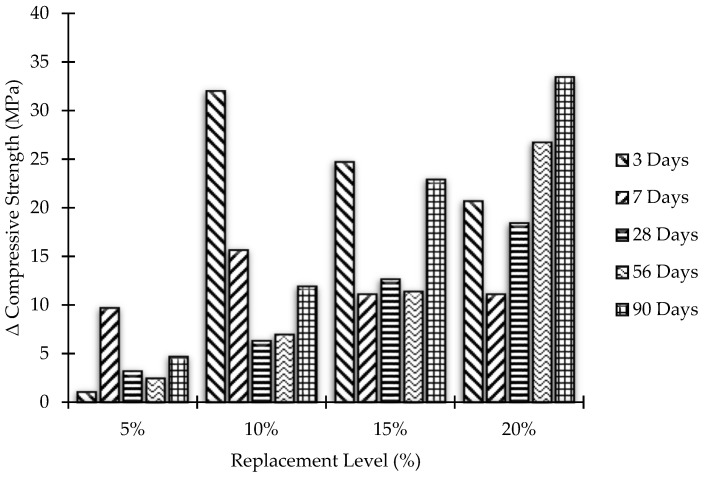
Compressive strength versus MK content.

**Figure 11:**
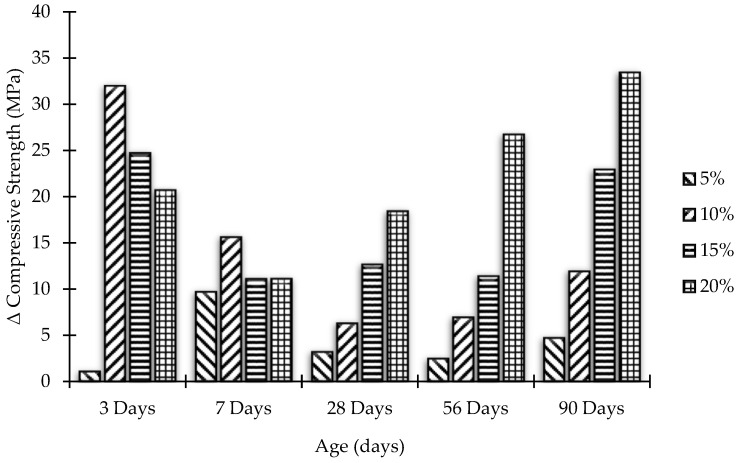
Effect of aging on compressive strength for different replacement levels of MK content.

Figure 8 and Figure 9 demonstrate the SAI of concrete samples after partial replacement of cement by MK, while Figure 10 and Figure 11 represent Δ increase/decrease in compressive strength of concrete using MK as pozzolanic material.

### 3.4. Water Permeability Test

**Figure 12:**
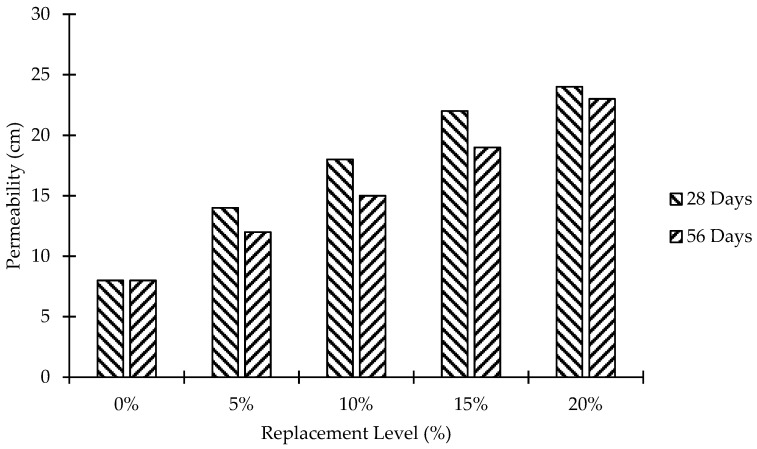
Replacement level versus permeability.

Permeability is a measurement of the flow of water under pressure in a saturated porous medium. For the water permeability test, six cubes (5.875 in. × 5.875 in. × 6 in.) were cast for each level of replacement. Samples were cast and cured in the same manner as for compressive strength testing. Three identical cubes were tested at 28 and 56 days of age in an automatic concrete permeabilizer (CN790). The results shown in Figure 12 demonstrated increased water penetration with increasing MK when compared to control concrete, which is in line with the findings of Nalawade et al. [13]. However, a relatively lesser water penetration was observed at 56 days as compared to permeability at 28 days against each replacement level of MK. The maximum value of water penetration was found at a 20% replacement level of MK, and it is 200% and 187.5% higher than that of control concrete at 28 days and 56 days, respectively. The increase in water permeability of MK blended concrete can be controlled by using different waterproofing techniques, for instance, by adding admixtures or water-reducing chemicals in the MK concrete mixes.

### 3.5. Sorptivity Test

**Figure 13:**
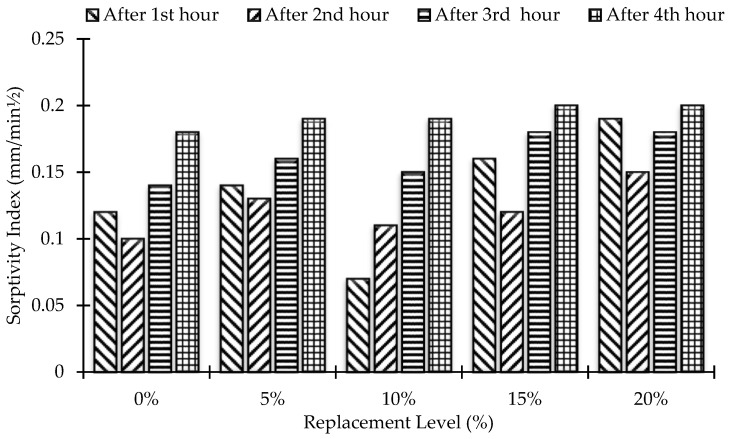
Replacement level versus sorptivity index up to 4 h.

**Figure 14:**
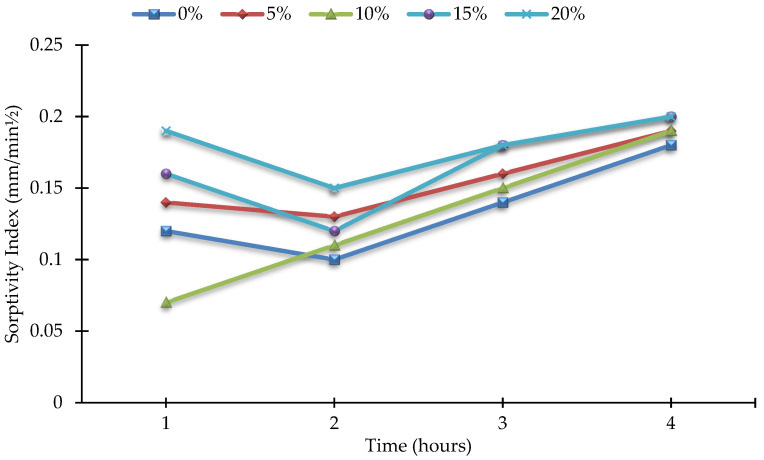
Rate of change of sorptivity index for all mixes.

Sorptiveness (S) is a material property that characterizes the tendency of porous materials to absorb and transport water by capillary action. For the sorptivity test, three identical cylinders (3.875 in. diameter, 2 in. height) were cast for each concrete mixture to calculate the average results. For test fluid, water was used, and the sorptivity of concrete was measured at age of 90 days. Figure 13 and Figure 14 show the comparison of sorptivity results among different concrete mixes. According to the results, the sorptivity of concrete increases with the increase in the amount of MK in the mix. In addition, the sorptivity index during the second hour was found to be less than that during the first hour for all the mixes, except for the 10% MK mix. The highest sorptivity was found for 20% MK concrete, and it is 11.11% higher than that of the control mix at a maximum duration of 4 h. The best sorptivity results were observed for 10% MK concrete, where the sorptivity index was the lowest, even lower than that of the control concrete during the first hour.

### 3.6. Water Absorption Test

**Figure 15:**
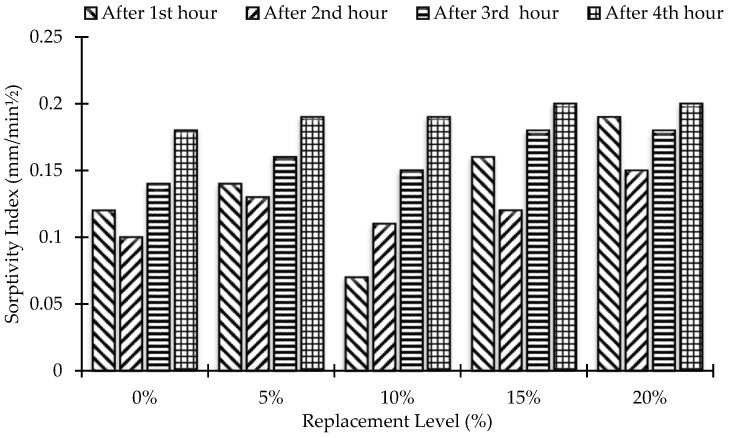
Replacement level versus percentage water absorption up to 4 h.

**Figure 16:**
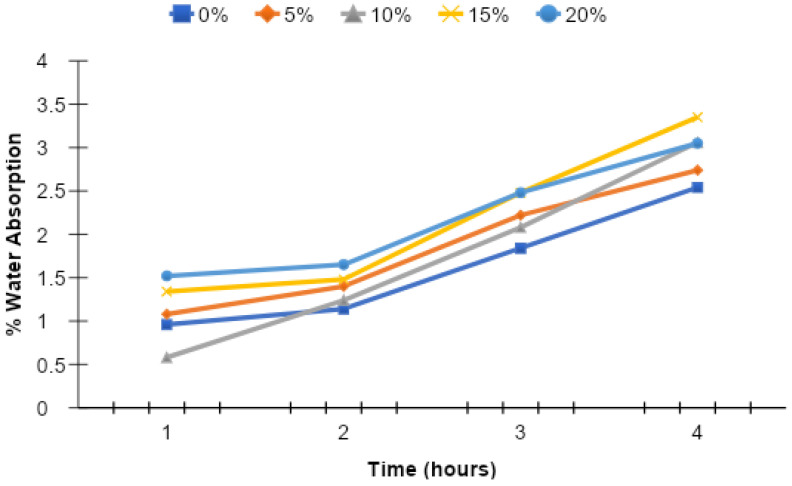
Rate of change of percentage water absorption for all mixes.

For the water absorption test, three cylinders (3.875 in. diameter, 2 in. height) were cast for each level of replacement. After casting, the samples were cured by soaking in water for 90 days. The water absorption rate of concrete was confirmed at the age of 90 days. The results are shown in Figure 15 and Figure 16. They show that as the equivalent value of MK increases, the water absorption of concrete increases. The maximum value of percentage water absorption was found at the 20% replacement level of MK and is 20.08% greater than that of the control mix at a maximum duration of 4 h. Water absorption for all samples was less than 3%, and thus they can be defined as good concrete [19].

### 3.7. Acid-Attacked Compressive Strength Test

**Figure 17:**
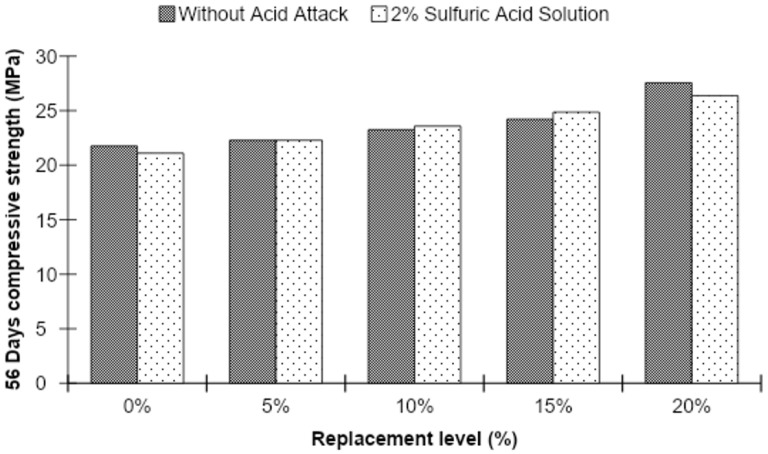
The 56-day compressive strength before and after acid attack for different MK contents.

**Figure 18:**
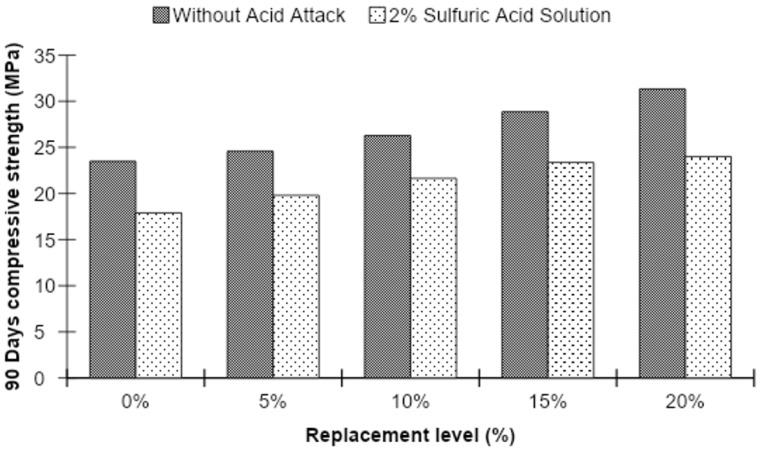
The 90-day compressive strength before and after acid attack for different MK contents.

Acid-attacked strength of concrete was checked at the ages of 56 and 90 days by performing a compressive strength test on MK blended concrete samples which were immersed in a 2% sulfuric acid solution after 28 days of normal curing. The compressive strength of acid-attacked concrete is demonstrated in Figure 17 and Figure 18.

Figures show that 56-day testing of concrete samples immersed in 2% sulfuric acid solution reflected a slight increase in compressive strength as compared to concrete cubes without acid attack at all replacement levels of MK except at 20%. However, an increase in compressive strength of the acid-attacked concrete is observed with increasing substitution levels of MK in the concrete, increasing by 25.02% over the control mixture.

However, the compressive strength of concrete cubes after 90 days of acid attack showed a similar decline at all MK levels compared to the strength of concrete cubes without acid attack. As the substitution level of MK in the concrete increases, the compressive strength of the acid-attacked concrete is observed to further increase, reaching a level 34.13% greater than that of the control mix.

### 3.8. Density of Acid-Attacked Concrete

**Figure 19:**
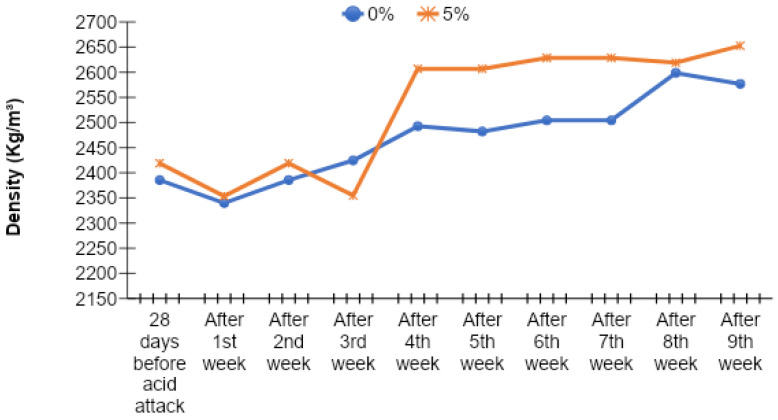
Comparison of density versus time curves for 0% (control) and 5% MK contents.

**Figure 20:**
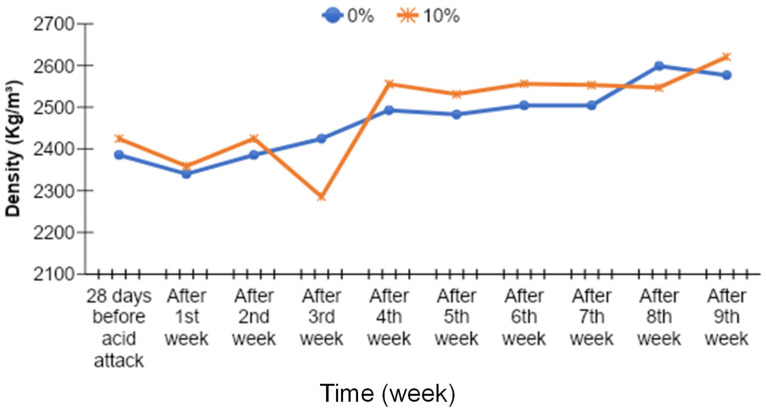
Comparison of density versus time curves for 0% (control) and 10% MK contents.

**Figure 21:**
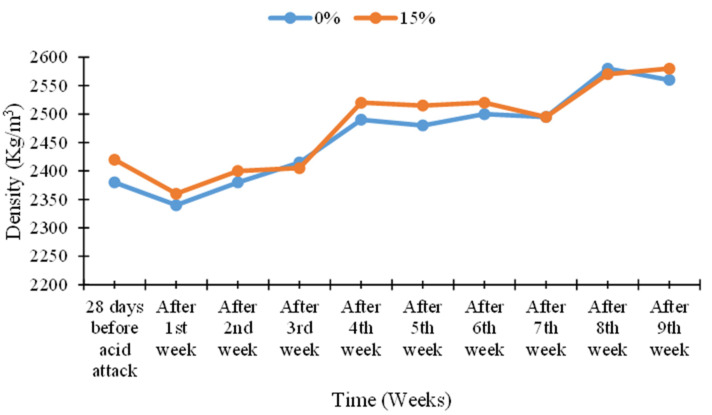
Comparison of density versus time curves for 0% (control) and 15% MK contents.

**Figure 22:**
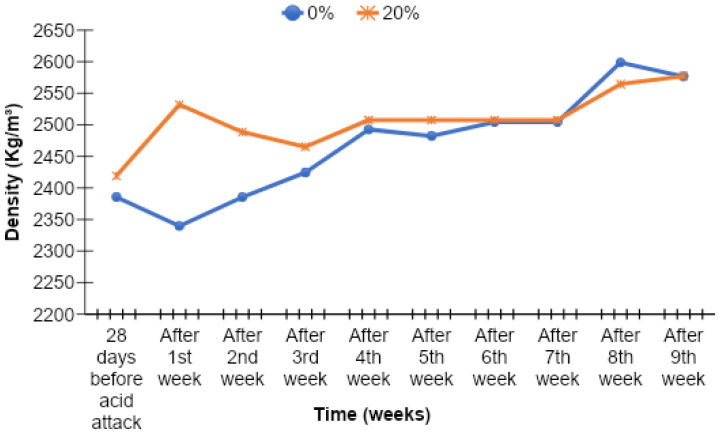
Comparison of density versus time curves for 0% (control) and 20% MK contents.

The density of acid-attacked concrete was checked at the age of 90 days. The density results of acid-attacked concrete were noted for the samples which were immersed in a 2% sulfuric acid solution after 28 days of normal curing. The density results of acid attack concrete with different percentages of cement replacement by MK are given in Figure 19, Figure 20, Figure 21 and Figure 22.

The density of acid-attacked concrete cubes increases with the increase in the replacement level of MK in concrete. Maximum density of acid-attacked concrete was achieved with a 5% replacement level of MK at the age of 90 days and is 2.94% greater than that of the control mix. On the other hand, at a 20% replacement level of MK for the same age, there is a 0.02% increase in density compared to the control mix.

Acid-attacked concrete also showed an increase in density with elapsed time. An increase in density with elapsed time for a 5% replacement level of MK by weight of cement after the ninth week was observed, reaching a level 12.70% greater than the density of acid-attacked concrete after first week. The initial decline after the first week was due to a lack of binding in concrete owing to the presence of extra absorbed water during curing which improved afterward over time. In this way, an increase in density with elapsed time for the 20% replacement level of MK by weight of cement after the ninth week was observed, as it was 1.78% greater than the density of acid-attacked concrete after the first week.

## 4. Conclusions and Recommendations

This study investigated the mechanical and durability performance of concrete incorporating different percentages of MK as a partial substitute for OPC. The following main conclusions were drawn from this study:

- Results revealed that the workability of concrete decreased gradually with the increase in the level of replacement of OPC with MK. The highest decrease in workability is up to 60% compared to that of control concrete and was observed for concrete containing 20% MK.
- Contrary to the workability performance, the compressive strength of concrete increased with increasing cement substitution with MK. A maximum increase of 33.43% in compressive strength was observed for concrete having 20% MK content at the age of 90 days when compared to control concrete at the same age.
- The water absorption percentage of hardened concrete increased with the increase in MK, although it remained within the permissible limits defined for good-quality concrete in the literature.
- Concrete samples immersed in 2% sulfuric acid solution did not show a significant decrease in compressive strength at 56 days as compared to the corresponding concrete without acid attack. On the other hand, the compressive strength of concrete at the age of 90 days after being subjected to acid attack showed a considerable decrease in comparison to the compressive strength of concrete not exposed to acid attack at the same age.
- In order to avoid laborious and costly testing of cement-replaced concrete related to mechanical and durability performance, data shall be collected from the available literature to develop advanced regression and machine learning models in accordance with the previous studies [20,21,22,23,24].

## Figures and Tables

**Table 1:** Physical properties of ordinary Portland cement (OPC).

Property	Value
Consistency (using Vicat apparatus)	29.75%
Initial setting time (using Vicat apparatus)	1 h and 50 min
Final setting time (using Vicat apparatus)	3 h and 57 min
Soundness (using Le Chatelier apparatus)	No expansion
Fineness (specific surface) (using Blain’s air permeability apparatus)	3128 cm^2^/g
Specific gravity	3.08

**Table 2:** Chemical composition of OPC.

Chemical Composition	% by Mass
SiO_2_	21.5
Al_2_O_3_	6.00
Fe_2_O_3_	3.75
CaO	62.0
SO_3_	2.80
MgO	2.75
K_2_O	1.00
Na_2_O	0.20
LOI	0.64

**Table 3:** Gradation of fine aggregates.

Sieve Size (BS)	Sieve Size (ASTM)	Mass Retained (g)	% Retained	Cumulative % Passing	Cumulative % Retained
9.5 mm	3/8-in	0	0	100	0
4.75 mm	# 4	19	1.90	98.1	1.90
2.36 mm	# 8	58	5.80	92.3	7.70
1.18 mm	# 16	138	13.8	78.5	21.5
600 μm	# 30	269	26.9	51.6	48.4
300 μm	# 50	373	37.3	14.3	85.7
150 μm	# 100	110	11.0	3.30	96.7
	Pan	33	3.30	0	-
	Total	1000 g		FM = 261.90/100 = 2.62	

**Table 4:** Gradation of coarse aggregate.

Sieve Size (BS)	Sieve Size (ASTM)	Mass Retained (g)	% Retained	Cumulative % Passing
25 mm	1 in	0	0	100
19 mm	¾ in	146	4.87	95.1
9.5 mm	3/8 in	1790	59.7	355
4.75 mm	3/16 in	984	32.8	2.67
Pan	Pan	80	2.67	-
		Total = 3000 g		

**Table 5:** Characteristics of mixing water.

Description	pH	Test Description		
		Hardness CaCo_3_ (mg/L)	Chloride (mg/L)	Sulfate SO_4_^−2^ (mg/L)
Obtained in this research	7.50	350	235	50
(Maximum allowable limit as per WHO guideline)	6.50–8.50	500	250	400

**Table 6:** Chemical composition of metakaolin.

Chemical Composition	% by Mass
Al_2_O_3_	38.9
SiO_2_	43.4
Fe_2_O_3_	2.67
CaO	2.74
MgO	0.20
SO_3_	0.28
Na_2_O	0.30
TiO_2_	0.79
Mn_2_O_3_	0.027

**Table 7:** Concrete mixture proportions.

Cement Replacement Level	Mixture ID	Mix Details
0%	MK0	100% OPC (Control Mix)
5%	MK1	95% OPC + 5% MK
10%	MK2	90% OPC + 10% MK
15%	MK3	85% OPC + 15% MK
20%	MK4	80% OPC + 20% MK

**Table 8:** Weights of the material used in casting specimens.

Cement Replacement	Mixture ID	Water (kg)	OPC (kg)	MK (kg)	Fine Aggregates (kg)	Coarse Aggregates (kg)
0%	MK0	20.6	37.5	-	75	150
5%	MK1	20.6	35.6	1.86	75	150
10%	MK2	20.6	33.8	3.72	75	150
15%	MK3	20.6	31.9	5.58	75	150
20%	MK4	20.6	30.0	7.44	75	150
Total for all mixes		103	168.9	18.6	375	750

## Data Availability

The data used in this research have been properly cited and reported in the main text.
